# Symptom patterns and health service use of women in early adulthood: a latent class analysis from the Australian Longitudinal Study on Women’s Health

**DOI:** 10.1186/s12889-023-15070-7

**Published:** 2023-01-21

**Authors:** Louise F. Wilson, Jenny Doust, Gita D. Mishra, Annette J. Dobson

**Affiliations:** School of Public Health, Faculty of Medicine, The University of Queensland, 288 Herston Road, Herston, QLD 4006 Australia

**Keywords:** Latent class analysis, Symptom profiles, Healthcare utilisation, Women, Australia

## Abstract

**Background:**

Symptoms can be strong drivers for initiating interaction with the health system, especially when they are frequent, severe or impact on daily activities. Research on symptoms often use counts of symptoms as a proxy for symptom burden, however simple counts don’t provide information on whether groups of symptoms are likely to occur together or whether such groups are associated with different types and levels of healthcare use. Women have a higher symptom burden than men; however studies of symptom patterns in young women are lacking. We aimed to characterise subgroups of women in early adulthood who experienced different symptom patterns and to compare women’s use of different types of health care across the different symptom subgroups.

**Methods:**

Survey and linked administrative data from 7 797 women aged 22–27 years in 2017 from the 1989–95 cohort of the Australian Longitudinal Study on Women’s Health were analysed. A latent class analysis was conducted to identify subgroups of women based on the frequency of 16 symptom variables. To estimate the associations between the latent classes and health service use, we used the “Bolck, Croon and Hagenaars” (BCH) approach that takes account of classification error in the assignment of women to latent classes.

**Results:**

Four latent classes were identified, characterised by 1) low prevalence of most symptoms (36.6%), 2) high prevalence of menstrual symptoms but low prevalence of mood symptoms (21.9%), 3) high prevalence of mood symptoms but low prevalence of menstrual symptoms, (26.2%), and high prevalence of many symptoms (15.3%). Compared to the other three classes, women in the high prevalence of many symptoms class were more likely to visit general practitioners and specialists, use more medications, and more likely to have had a hospital admission.

**Conclusions:**

Women in young adulthood experience substantially different symptom burdens. A sizeable proportion of women experience many co-occurring symptoms across both physical and psychological domains and this high symptom burden is associated with a high level of health service use. Further follow-up of the women in our study as they enter their late 20 s and early 30 s will allow us to examine the stability of the classes of symptoms and their associations with general health and health service use. Similar studies in other populations are needed to assess the generalisability of the findings.

**Supplementary Information:**

The online version contains supplementary material available at 10.1186/s12889-023-15070-7.

## Background

Individuals present to the healthcare system when they have symptoms of sufficient frequency, duration or severity [1, 2]. Most studies assessing the relationship between symptoms and use of health care services have used symptom counts as a proxy for symptom burden [1–3]. While useful, simple counts do not provide information on whether groups of symptoms are likely to occur together in distinct patterns or clusters, or whether such groups are associated with different types and levels of healthcare utilisation.

One statistical approach that can be used to identify complex symptom patterns is latent class analysis. Latent class analysis is a person-centred technique that can be used to classify individuals in a heterogenous study population into more homogenous subgroups based on a set of observed categorical variables [4].

Overall, women report a higher symptom burden than men across all age groups [1–3]. Latent class analysis of somatic symptoms in a Danish population found that women had a higher probability than men of being in classes with many symptoms compared with the class characterised by no symptoms [5]. Latent class analysis has been used to identify symptom patterns in women; however, these studies have either focussed on narrow groups of symptoms (e.g., menopausal [6, 7], mood [8], menstrual and mood [9]), symptom patterns among women with specific conditions (e.g., dysmenorrhea [10], irritable bowel syndrome [11]) or women in midlife (i.e., during menopause transition) [12–14].

To our knowledge no study has evaluated patterns among a broad group of recurring symptoms in a community-based sample of women in the early adulthood, or how different patterns may be associated with differences in health service use. We had the opportunity to undertake such a study using data from a cohort of women born in 1989–95 participating in Australian Longitudinal Study on Women’s Health.

Therefore, the purpose of the current study was to model latent classes of symptoms experienced by women in early adulthood and to compare women’s use of different types of health care based on the symptom classes. We hypothesised that there would be distinct latent classes of young women who experienced different symptoms and that these subgroups would differ significantly in healthcare use.

## Methods

### Study design and participants

This is an observational cohort study using self-report survey and linked administrative data from the 1989–95 cohort of the Australian Longitudinal Study on Women’s Health (ALSWH). The ALSWH is a national longitudinal study established to investigate factors contributing to women’s health and wellbeing and their use of health services across key life stages. The study began in 1996 when three cohorts of women born in 1973–78, 1946–51 and 1921–26 were included in the study [15]. In 2012–13, a fourth cohort of women born in 1989–95 were recruited to provide contemporary information about women in early adulthood. Eligibility and recruitment methods have been described in detail elsewhere [16, 17]. Women in the 1989–95 cohort were surveyed annually between 2013 and 2017 (Surveys 1 to 5) and in 2019 (Survey 6). We analysed self-report data from the most recent survey with at least 12 months of linked administrative data following return of the survey, that is Survey 5 (2017, when the women were aged 22–27 years).

### Symptom variables

The survey question on common symptoms was first used in the baseline surveys of the original cohorts enrolled in ALSWH in 1996. The list differed slightly across the cohorts to reflect symptoms that were age-specific (e.g., menstrual and menopause symptoms). The symptom lists were developed and finalised following nine focus groups and five pilot studies conducted prior to the baseline surveys that explored survey methods and content, including the frequency distribution of responses [18, 19].

The survey asked women if they had experienced the following symptoms in the past 12 months with response options of ‘never’, ‘rarely’, ‘sometimes’ or ‘often’: allergies, headaches/migraines, severe tiredness, back pain, vaginal discharge or irritation, premenstrual tension, irregular periods, heavy periods, severe period pain, skin problems, difficulty sleeping, depression, episodes of intense anxiety, palpitations, urine that burns or stings, leaking urine, constipation, haemorrhoids, other bowel problems. Due to the low prevalences of ‘often’ responses for the two urinary symptoms and the haemorrhoid symptoms, we combined the responses for ‘urine that burns or stings’ and ‘leaking urine’ into ‘any urinary symptoms’, and responses for ‘constipation’, ‘haemorrhoid’ and ‘other bowel problems’ into ‘any bowel symptoms’. If a woman responded that she had not experienced either urine that burns or stings or leaking urine, she was categorised as never experiencing any urinary problems; of the remaining women, if the response was ‘often’ for one of urine that burns or stings or leaking urine then a woman was categorised as often experiencing any urinary problems. Of the remaining women, a response of ‘sometimes’ for either urine symptom variable was categorised as ‘sometimes’; lastly women were categorised as ‘rarely’ experiencing any urinary symptoms. Similarly for bowel symptoms.

As we were most interested in women who reported experiencing symptoms more frequently (i.e. sometimes or often), and to facilitate the interpretation of the latent classes, we created three-category symptom variables for use in the latent class analysis, combining the ‘never’ and ‘rarely’ response options (‘never/rarely’, ‘sometimes’, ‘often’).

### Healthcare utilisation

We sourced information on general practitioner (GP) visits, specialist visits, medication use, and same day and overnight hospital admissions through data linkage with health administrative databases.

The Australian Institute of Health and Welfare (AIHW) conducts record linkage and extraction for the Medical Benefits Schedule (MBS) and Pharmaceutical Benefits Scheme (PBS). Medicare Personal Identifier Numbers (PINs) for ALSWH participants were validated by Medicare Australia on enrolment to the Study. The AIHW conducts annual deterministic data linkage of ALSWH cohorts, using the Medicare PINs. Checks are also undertaken periodically to investigate any apparent discrepancies. Therefore, the sensitivity of matching for these datasets is considered extremely high. Researchers only have access to de-identified participant data.

#### General practitioner and specialist visits

We counted the number of general practitioner (GP) and specialist visits participants had in the 12 months after completing Survey 5 using linked MBS data for GP visits (using the most common MBS item numbers billed by GPs: #3, #4, #23, #24, #36, #37, #44, #47) and the number of MBS items billed in the category Broad Type of Service MBS Item number #0200 for specialist visits (not including obstetrics). We categorised the count of GP visits into “less than 2 visits”, “2 to 3 visits”, “4 to 6 visits”, “7 to 9 visits”, “10 to 12 visits” and “More than 12 visits”. The count of specialist visits was categorised as “No visits”, “1 to 2 visits” and “3 or more visits”.

#### Prescription medication use

Information on medication prescriptions filled was obtained from the PBS datasets. We counted the number of unique prescribed medicines (identified by the Anatomic Therapeutic Chemical (ATC) classification system at the fifth level i.e., chemical substance level [20]) dispensed during 1 April – 30 September 2017. We used this timeframe to avoid the ‘safety net effect’ [21]. The PBS includes a safety net threshold to limit the out-of-pocket costs individuals and families spend on prescription medicines in a calendar year [22]. Once the threshold is reached in a given year, prescriptions are heavily discounted or free for the remainder of that calendar year, resulting in stockpiling of medicines at the end of year and filling of fewer prescriptions at the beginning of the following year as the stockpile is used [21]. Hence the choice of time period 1 April to 30 September. We categorised the count of medicines as “no prescriptions”, “1 prescription”, “2 prescriptions”, “3 or more prescriptions”.

#### Hospital admissions

In Australia, hospital admissions data are maintained at the State and Territory level. ALSWH data are linked with hospital data using probabilistic linkage based on name, date of birth, address, and address history. For New South Wales, Victoria, Queensland and Western Australia, public and private hospital records are included in the data collection; for the Australian Capital Territory, Northern Territory, South Australia, and Tasmania data on nominated public hospitals only are included, therefore undercounting of hospital admissions by participants residing in these jurisdictions may have occurred. We created a dichotomous variable indicating whether the participant had a hospital admission of any type (same day or overnight) in the 12 months after completing Survey 5. We also created two separate dichotomous variables for same-day and overnight hospital admission. Women who had a hospital admission with a pregnancy-related primary diagnosis (ICD-10 codes O00-O9A) were not counted as having a hospital admission.

### Covariates

All the covariates included in the analysis were self-reported at Survey 5. Sociodemographic variables were: age at Survey 5 (continuous variable), area of residence (major cities, inner regional, outer regional/remote/very remote [23]), ability to manage on available income (not too bad/easy, difficult sometimes, difficult all the time), highest qualification level (university degree or higher, certificate/diploma, high school or less), partner status (engaged/married/living with partner, has partner but not living together, single), and number of children (none, one child, two or more children). Behavioural variables were current smoking status (yes, no), frequency of heavy episodic drinking i.e., five or more drinks on one occasion (less than once a month, at least once a month), cannabis use in the last 12 months (yes, no), and level of physical activity (none/low level – < 150 min per week, moderate/high level – at least 150 min per week [24]). Body mass index (BMI) [25] was calculated from self-reported height and weight and categorised as < 25 kg/m^2^, 25–29.9 kg/m^2^, ≥ 30 kg/m^2^).

### Statistical analysis

#### Latent class analysis

We used latent class analysis to identify groups of participants with different patterns of symptoms. We did not have an a priori hypothesis on the assignment of symptoms to classes or the number of latent classes. Models with one through to six classes were fitted using the expectation–maximization (EM) algorithm [26] with 100 replicates of the analysis based on different sets of random starting values. To select the model with the optimal number of classes we used the following model fit criteria: Akaike information criterion (AIC) [27] and Schwarz Bayesian information criterion (BIC) [28] with lower values indicating a better balance between model fit and model parsimony; the degree of separation of the latent classes using the entropy statistic which measures the overlap of classes—with higher entropy values indicating better separation) [29]; relative class sizes [30]; model stability (with higher percentage of replicates associated with best-fitting model indicating good model stability) and the substantive meaningfulness of the model [31]. Finally, in the model selected, we present the average posterior probabilities (AvePP) of class membership (each individual is assigned a posterior probability of membership in each latent class given their specific profile of measured symptoms) in a matrix to assess the accuracy of correct classification to a latent class (with diagonal AvePPs > 0.7 indicating well-separated latent classes) [32]. To name and characterise the latent classes in the best-fitted model we compared the percents for both the ‘sometimes’ and ‘often’ categories across all symptoms in the total study population with the corresponding percents in each of the latent classes.

#### Factors associated with latent class membership

We described the sociodemographic, health and behaviour characteristics of the study population and by latent class status. We used multinomial logistic regression to estimate odds ratios (OR) and 95% confidence intervals (CI) for the associations between the sociodemographic, health and behavioural factors and the symptom latent classes using the “Bolck, Croon and Hagenaars” (BCH) method to take account of classification error in the assignment of women to latent classes (the BCH approach [33]).

To estimate the latent classes, an EM algorithm is used that estimates missing values on the assumption that data is missing at random [31]. To estimate the associations between the covariates and the latent classes, multinomial logistic regression is used to estimate coefficients that are used in the calculation of the odds ratios and inverse propensity weights. This part of the analysis used complete case data. To ensure that the estimation of the latent classes and the regression analyses were done on the same group of women, we removed participants with missing data on any of the covariates before fitting the latent classes (we had complete follow-up information on health service use).

#### Latent classes and health service use

Due to the uncertainty in the direction of the associations between covariates and symptoms (i.e., certain sociodemographic, health and behaviour factors may cause some symptoms; equally some symptoms may have an impact on certain sociodemographic, health and behaviour factors, or the associations may be bi-directional), in our primary analysis we estimated univariate associations between symptom latent class membership and health service use using only the BCH approach that takes into account the latent class uncertainty. For completeness, we additionally used inverse propensity (IP) weights [34] to balance the effects of differences in sociodemographic, health and behavioural factors between the latent classes. Further details on these methods can be found in Additional File 1: Appendix S1.

All analyses were done using SAS software, Version 9.4 (TS1M6) of the SAS system for Windows copyright © 2016 by SAS Institute Inc (Carey). We used PROC LCA [31] to identify the latent classes and the SAS %LCA_Covariates_3Step macro [35] to estimate the associations between covariates and latent classes and estimate the IP weights. The SAS %LCA_Distal_BCH macro [36] was used to estimate the associations between the latent classes and different types of health service use.

## Results

Survey 5 was completed by 8 495 participants. We included 7 797 women in our analysis; in total 698 women were excluded due to missing data on all symptoms (*n* = 25) or any of the covariates (*n* = 673; < 4% missing data on any one covariate).

**Fig. 1:**
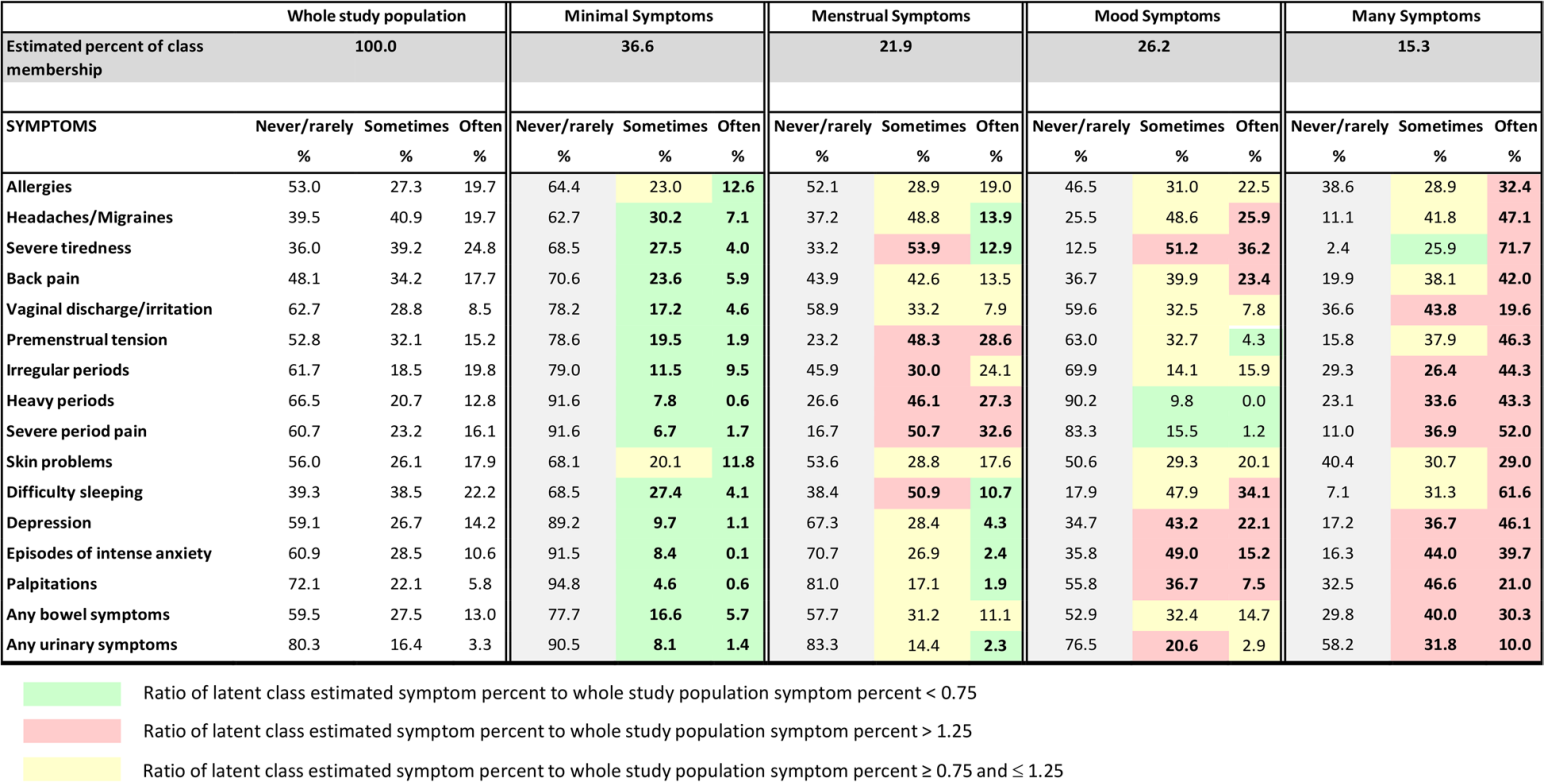
Percent of symptom frequency for whole study population and percent of symptom frequency by latent class status – four class model (*N* = 7797)

In the analysis sample, only 2% of women reported that they never/rarely experienced all the symptoms (i.e., were relatively symptom-free). Ninety-six percent of women reported they sometimes experienced at least one symptom; while 22% of women reported they sometimes experienced 7 or more symptoms. The most common symptoms women reported experiencing sometimes were headaches/migraines (41%), severe tiredness (39%), difficulty sleeping (39%), back pain (34%) and premenstrual tension (32%) (Fig. 1). Seventy-six percent of women reported they often experienced at least one symptom; while 7% of women reported they often experienced 7 or more symptoms. The most common symptoms that women reported experiencing often were severe tiredness (25%), difficulty sleeping (22%), allergies (20%), headaches/migraines (20%) and irregular periods (20% (Fig. 1)).

### Latent class identification

Based on the sixteen symptom variables, a model with four latent classes was considered to provide the best fit. Model fit criteria are summarised in Table 1. Although there were small reductions in the AIC and BIC in the 5- and 6-class models compared to the 4-class model, both entropy and model identification were better for the 4-class model. In addition, the 4-class model had average posterior probabilities on the diagonal of > 0.8 for all latent classes indicating well-separated classes (Table 2).

**Table 1:** Goodness of Fit statistics for 1 to 6 latent class models

Number of classes	Degrees of Freedom	AIC	BIC	Log-likelihood	Smallest class size probability	Entropy	% replicates producing the best-fitting model
1	43,046,688	98,005.15	98,227.92	-116,021.76	1.00	1.00	100
2	43,046,655	84,458.50	84,911.00	-109,215.43	0.43	0.80	100
3	43,046,622	81,831.43	82,513.66	-107,868.90	0.25	0.78	45
**4**	**43,046,589**	**79,517.29**	**80,429.24**	**-106,678.82**	**0.15**	**0.77**	**100**
5	43,046,556	78,732.52	79,874.21	-106,253.44	0.11	0.74	65
6	43,046,523	78,120.30	79,491.72	-105,914.33	0.06	0.73	61

*Abbreviations: AIC* Akaike’s Information Criterion, *BIC* Bayesian Information Criterion

**Table 2:** Average posterior probabilities of class membership—four-class model

Latent class	1	2	3	4
1. Minimal Symptoms	**0.905**	0.038	0.056	0.000
2. Menstrual Symptoms	0.053	**0.842**	0.059	0.046
3. Mood Symptoms	0.067	0.056	**0.840**	0.038
4. Many Symptoms	0.000	0.065	0.044	**0.891**

Diagonal values are the average posterior probability of a women being assigned to a class in the four-class model given her specific symptom pattern. Off-diagonal values are the average posterior probability of being assigned to another class in the four-class model. Average posterior probabilities > 0.7 on the diagonal indicate a well-separated latent class model

The proportion of women in each of the four latent classes and the percentages of symptom frequency by latent class status are shown in Fig. 1. For brevity, we named the four classes Minimal Symptoms, Menstrual Symptoms, Mood Symptoms and Many Symptoms. The four latent classes were distinguished by the following characteristics.

#### Minimal  Symptoms (36.6%)

Probability of membership of this class was characterised by:

- percents for both the ‘sometimes’ and ‘often’ categories across all symptoms that were lower than the percents for the whole study population (marginal percents).

#### Menstrual Symptoms (21.9%).

Probability of membership of this class was characterised by:

- higher (than marginal) percents for ‘sometimes’ and ‘often’ reporting menstrual symptoms (premenstrual tension, heavy periods, severe period pain) and ‘sometimes’ reporting irregular periods, severe tiredness and difficulty sleeping; and
- lower (than marginal) percents for ‘often’ reporting headaches/migraines, severe tiredness, difficulty sleeping, depression, episodes of intense anxiety, palpitations, and any urinary problems.

#### Mood Symptoms (26.2%)

Probability of membership of this class was characterised by:

- higher percents for ‘sometimes’ and ‘often’ reporting severe tiredness, depression, episodes of intense anxiety and palpitations, ‘often’ reporting headaches/migraines, back pain, difficulty sleeping and ‘sometimes’ reporting any urinary problems; and
- lower percents for ‘sometimes’ or ‘often’ reporting heavy periods and ‘often’ reporting premenstrual tension and severe period pain.

#### Many Symptoms (15.3%)

Probability of membership of this class characterised by:

- higher percents for the ‘often’ categories across all symptoms.

### Factors associated with latent class membership

The sociodemographic, health and behaviour characteristics of the study population and by latent class status are described in Table 3. There were differences in these characteristics across latent classes. Table 4 shows the associations between sociodemographic, behavioural and health factors and the probability of being in the Menstrual, Mood and Many Symptoms classes compared to the Minimal Symptoms class. Using the BCH approach and adjusting for all other covariates in the model, experiencing difficulties managing on income was the factor most strongly associated with the membership of Mood Symptoms and Many Symptoms. Being obese was associated with two-fold and three-fold odds of being in Menstrual Symptoms and Many Symptoms respectively (Table 4). Current smokers, recent cannabis users and women with education levels less than a bachelor’s degree were also more likely to be in the Menstrual, Mood and Many Symptoms classes (Table 4).

**Table 3:** Sociodemographic, behavioural and health factors at Survey 5 for whole study population (*N* = 7797) and by latent class membership

**Covariates**	**Whole study population**	**Minimal Symptoms** **(36.6%)**	**Menstrual Symptoms** **(21.9%)**	**Mood Symptoms** **(26.2%)**	**Many Symptoms** **(15.3%)**
	**Percent**	**Percent**^**a**^	**Percent**^**a**^	**Percent**^**a**^	**Percent**^**a**^
***Area of residence***					
Major cities	75.2	75.2	73.7	77.5	73.4
Inner regional	16.5	16.8	16.2	15.0	18.6
Outer regional/remote/very remote	8.3	8.0	10.1	7.5	8.0
***Highest education level***					
High school or less	15.5	11.0	13.0	18.0	25.5
Certificate/Diploma	25.5	16.1	26.0	29.0	41.5
Degree or higher	59.0	72.9	61.0	53.0	33.0
***Ability to manage on income***					
Not too bad/easy	52.6	70.1	53.3	42.9	26.4
Sometimes difficult	31.8	23.8	35.5	36.8	36.7
Always difficult	15.6	6.1	11.2	20.3	36.9
***Partner status***					
Engaged/married/living with partner	49.2	50.9	50.1	48.0	45.7
Has a partner, not living together	21.4	22.0	22.4	19.8	21.5
Single	29.4	27.1	27.5	32.2	32.8
***Number of children***					
None	89.7	91.9	91.6	87.4	85.6
One child	6.1	5.1	5.0	6.9	8.5
Two or more children	4.2	3.0	3.4	5.7	5.9
***Heavy episodic drinking***					
Less than once a month	73.7	72.7	74.2	73.5	75.9
At least once a month	26.3	27.3	25.8	26.5	24.1
***Cannabis use***					
Has not used in last 12 months	70.0	77.4	68.8	67.2	59.0
Has used in last 12 months	30.0	22.6	31.2	32.8	41.0
***Current smoking status***					
No	85.4	92.5	86.2	83.5	70.7
Yes	14.6	7.5	13.8	16.5	29.3
***Level of physical activity***					
< 150 min per week	30.2	22.9	28.3	35.4	41.1
≥ 150 min per week	69.8	77.1	71.7	64.6	58.9
***Body mass index (BMI)***					
< 25.0 kg/m^2^	56.6	66.8	53.4	54.3	40.9
25.0 to 29.9 kg/m^2^	23.3	21.8	25.5	24.3	21.9
≥ 30 kg/m^2^	20.1	11.4	21.1	21.4	37.2
	**Mean**	**Mean**	**Mean**	**Mean**	**Mean**
***Age (continuous)***	24.6	24.8	24.6	24.6	24.4

^a^Estimated percents are weighted to account for uncertainty in class assignment

**Table 4:** Adjusted odds ratios (OR) and 95% confidence intervals (CI) for the associations between sociodemographic, health and behavioural factors and symptom latent classes in the 1989–95 cohort of the Australian Longitudinal Study on Women’s Health (*n* = 7779) at Survey 5 (2017, aged 22–27 years)

Covariates	**Minimal Symptoms (36.6%) versus**		
	**Menstrual Symptoms (21.9%)**	**Mood Symptoms (26.2%)**	**Many Symptoms (15.3%)**
	**OR (95% CI)**^**a**^	**OR (95% CI) **^**a**^	**OR (95% CI) **^**a**^
***Area of residence***			
Major cities	Ref	Ref	Ref
Inner regional	0.96 (0.78, 1.18)	0.80 (0.65, 0.99)	0.95 (0.75, 1.21)
Outer regional/remote/very remote	1.28 (0.98, 1.68)	0.86 (0.64, 1.15)	0.91 (0.65, 1.26)
***Highest education level***			
Degree or higher	Ref	Ref	Ref
Certificate/Diploma	1.51 (1.24, 1.84)	1.71 (1.41, 2.08)	2.90 (2.33, 3.61)
High school or less	1.11 (0.86, 1.42)	1.54 (1.23, 1.93)	2.60 (2.04, 3.61)
***Ability to manage on income***			
Not too bad/easy	Ref	Ref	Ref
Sometimes difficult	1.75 (1.48, 2.07)	2.13 (1.80, 2.52)	2.90 (2.35, 3.57)
Always difficult	1.94 (1.45, 2.60)	4.05 (3.14, 5.22)	8.81 (6.77, 11.45)
***Partner status***			
Engaged/married/living with partner	Ref	Ref	Ref
Has a partner, not living together	1.04 (0.85, 1.27)	0.96 (0.78, 1.18)	1.07 (0.85, 1.36)
Single	0.96 (0.80, 1.15)	1.19 (0.99, 1.42)	1.11 (0.90, 1.37)
***Number of children***			
None	Ref	Ref	Ref
One child	0.71 (0.49, 1.02)	0.98 (0.70, 1.36)	0.92 (0.65, 1.31)
Two or more children	0.81 (0.52, 1.28)	1.26 (0.85, 1.86)	0.90 (0.58, 1.41)
***Heavy episodic drinking***			
Less than once a month	Ref	Ref	Ref
At least once a month	0.84 (0.70, 1.01)	0.90 (0.75, 1.07)	0.76 (0.61, 0.94)
***Cannabis use***			
Has not used in last 12 months	Ref	Ref	Ref
Has used in last 12 months	1.44 (1.20, 1.73)	1.44 (1.21, 1.73)	1.74 (1.42, 2.13)
***Current smoking status***			
No	Ref	Ref	Ref
Yes	1.51 (1.15, 1.97)	1.57 (1.22, 2.02)	2.60 (2.02, 3.36)
***Level of physical activity***			
< 150 min per week	Ref	Ref	Ref
≥ 150 min per week	0.83 (0.69, 0.98)	0.61 (0.52, 0.71)	0.56 (0.46, 0.68)
***Body mass index (BMI)***			
< 25.0 kg/m^2^	Ref	Ref	Ref
25.0 to 29.9 kg/m^2^	1.40 (1.16, 1.68)	1.23 (1.03, 1.48)	1.41 (1.13, 1.76)
≥ 30 kg/m^2^	2.01 (1.61, 2.51)	1.68 (1.35, 2.09)	3.27 (2.60, 4.10)
***Age (per year older)***	0.98 (0.94, 1.02)	0.98 (0.94, 1.03)	0.92 (0.88, 0.97)

^a^All variables listed in the table are included in the model

### Latent classes and health service use

**Fig. 2:**
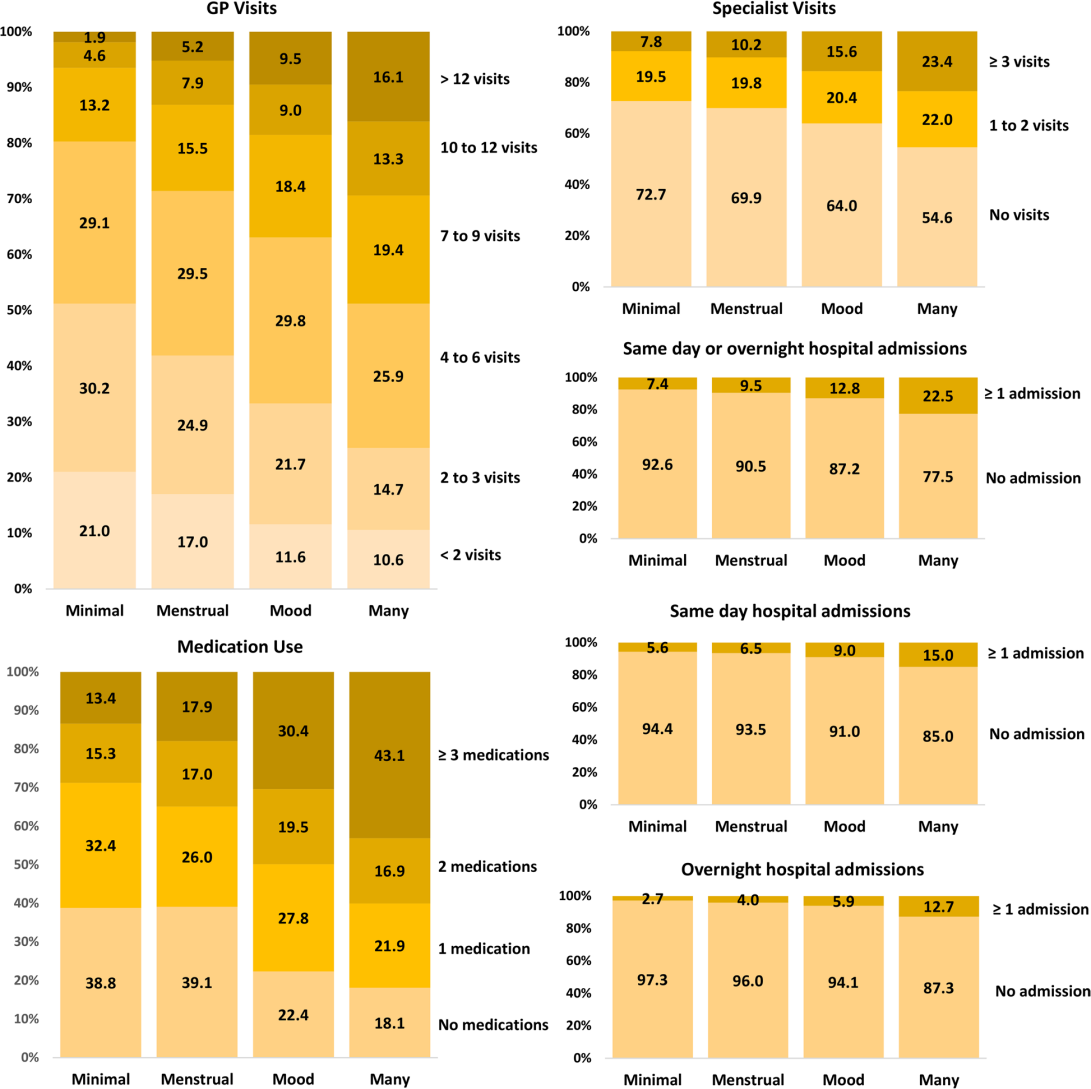
Proportions for different types of health service use ( weighted to account for uncertainty in class assignment) by symptom latent class status in women aged 22–27 years at Survey 5 in the 1989–95 cohort of the Australian Longitudinal Study on Women’s Health (*N* = 7797). Percent of women in each latent class: Minimal Symptoms (36.6%), Menstrual Symptoms (21.9%), Mood Symptoms (26.2%), Many Symptoms (15.3%)

Figure 2 shows the BCH-weighted percentages in the categories of each health care type across the four latent classes. For all types of health care, use was highest in Many Symptoms, followed by Mood Symptoms and Menstrual Symptoms, and lowest for Minimal Symptoms (Fig. 2). Sixteen percent of women in Many Symptoms had more than 12 GP visits in the 12 months after self-report of symptoms, 43% filled at least three prescriptions for different medications, 23% had at least three specialist visits, and 22% had at least one same day or overnight hospital admission; by comparison the equivalent percentages for Minimal Symptoms were 2% (≥ 12 GP visits), 13% (≥ 3 medications), 8% (≥ 3 specialist visits) and 7% (≥ 1 hospital admission) (Fig. 2). After IP weighting, the classes were evenly balanced with respect to the included covariates (Additional File 1: Figure S1). Nevertheless, there was minimal difference between the BCH-weighted and BCH- and IP-weighted associations between symptom class membership and health service use (Additional File 1: Table S1).

## Discussion

We found that women in young adulthood can experience substantially different symptom burdens. Nearly all women reported that they had experienced at least one of sixteen symptoms in the preceding 12 months. The most reported symptoms were severe tiredness, difficulty sleeping and headaches/migraines. Four distinct symptom classes were identified, characterised by low prevalence of most symptoms (37% of women), high prevalence of menstrual symptoms but low prevalence of mood symptoms (22%), high prevalence of mood symptoms but low prevalence of menstrual symptoms (26%), and high prevalence of many symptoms (15%). Not unexpectedly, women in the latent class characterised by a high prevalence of many symptoms visited GPs and specialists more often, used more medications, and were the most likely to have same day and overnight hospital admissions. Income difficulties and obesity were the factors associated most strongly with the more symptomatic classes (particularly the Mood and Many symptom classes); however weighting for these and other covariates did not attenuate the associations with higher use of health services by women in these classes.

Population-based studies using counts of symptoms have all found, similar to our study, that only a small minority of participants report no symptoms [3, 37, 38] even in younger age groups [38]. In our study, the most reported symptoms were severe tiredness, difficulty sleeping and headaches/migraines. This finding is congruent with both a New Zealand study (of adult men and women of all ages) that measured the severity of a list of 46 acute vomiting) and commonly recurring symptoms (e.g. headaches, indigestion) in the past seven days and found fatigue, back pain and headache were the most common symptoms reported by women [3], and a Swedish study where the most commonly reported symptoms in the previous 3 months reported by women (aged 25–99 years) from a list of 30 general symptoms (with yes/no response options) were fatigue, headaches, melancholy and back pain [37].

To our knowledge, this is first study that has looked at patterns of a broad range of recurring symptoms in women in early adulthood. Although not directly comparable due to differences in the characteristics of study populations, symptom lists and timeframes over which symptoms are reported, our finding of a latent class with minimal symptoms and a latent class with many symptoms is consistent with studies of symptom patterns in populations of both men and women across broad age groups [5, 39], women in mid-life [7, 13], and cancer patients [40].

In addition to these two classes, we identified two latent classes characterised by high prevalence of menstrual symptoms (but low prevalence of depression and anxiety symptoms) and high prevalence of mood symptoms (but low prevalence of menstrual symptoms). While premenstrual tension [41], dysmenorrhea [42] and heavy menstrual bleeding [43] are all associated with symptoms of depression and anxiety, our study indicates that there are groups of women who do not experience mood and menstrual symptoms together. Our findings are consistent with a Chinese study that used latent class analysis to identify patterns of premenstrual syndrome (PMS) and depression symptoms in university students and found that there were groups of women who experienced PMS and depressive symptoms separately [9]. While the Menstrual symptoms and Mood symptoms latent classes were characterised by menstrual and mood symptoms respectively, the reported frequency of these symptoms was more likely to be ‘sometimes’ than ‘often’ compared to the Many symptoms class, and thus may at least partly explain the difference in health service use across these three classes.

Strengths of this study include the large community-based sample of young women, the low amount of missing data on the symptom variables, and the completeness of the GP, specialist and prescription medication use data. In addition, self-report of symptoms captured the experience of all of the community-based sample and not just those who sought formal health care for their symptoms.

Limitations are that all the covariate information was self-reported which may introduce biases, such as recall and social desirability bias, into the analysis. Our latent class analysis was exploratory in nature with the results contingent upon the list of symptoms and the characteristics of the study sample. Our symptoms list was not based on an established survey instrument but was derived through multiple focus groups and pilot testing and represents a broad range of symptoms commonly reported by young Australian women. Comparative research of symptom burden is limited by the absence of a commonly used measure of symptoms. A systematic review of self-report symptom questionnaires used in large-scale studies identified 40 different questionnaires that varied in length, the time-frame used for recall of symptoms and whether frequency and/or severity of symptoms was measured [44]. We do not know if the health service use was directly related to the reported symptoms, or which symptoms or groups of symptoms may have precipitated contact with the health system. There may have been some under-counting of hospital admissions for women living in the Northern Territory, Australian Capital Territory, South Australia, or Tasmania as private hospital admissions were not included. However, this number is likely to be fairly small as these are the least populous jurisdictions with approximately 15% of study participants living in these states/territories at the time of completing Survey 5. We also did not have information on use of over-the-counter medication or complementary health care by women. Finally, while generally representative, compared with the female population at the 2016 Australian Census, our study sample was more educated than all Australian women aged 22–27 years (59% with a bachelor degree or higher compared to 38% [45]), more likely to be born in Australia (92% compared to 70% [46]), and speak English at home (97% compared to 71% [47]).

Further follow-up of the women in our study as they enter their late 20 s and early 30 s will allow us to look at the stability of the latent classes and class membership over time, and if symptom class membership is associated with general health and health service use. Similar studies of symptoms experience by women at similar life stages in other populations would increase understanding of the nature and impact of symptom burden on women’s well-being.

## Conclusions

This study demonstrates that women in young adulthood experience substantially different symptom burdens, that a sizeable proportion of women experience many co-occurring symptoms across both physical and psychological domains and that high symptom burden is associated with a high level of health service use.

## Supplementary Information

**Additional file 1: Appendix S1.** The BCH approach and calculation of Inverse Propensity Weights. **Figure S1.** Covariate balance plot [standardised difference of BCH-weighted and BCH- and Inverse propensity weight-adjusted distribution] for Menstrual, Mood and Many Symptoms groups compared to Minimal Symptoms group. **Table S1.** BCH-weighted and BCH- and inverse propensity (IP) weighted percents of health case use for the whole study population and by symptom latent classes.

## Data Availability

ALSWH survey data are owned by the Australian Government Department of Health and Aged Care and due to the personal nature of the data collected, release by ALSWH is subject to strict contractual and ethical restrictions. Ethical review of ALSWH is by the Human Research Ethics Committees at The University of Queensland and The University of Newcastle. De-identified data are available to collaborating researchers where a formal request to make use of the material has been approved by the ALSWH Data Access Committee. The committee is receptive of requests for datasets required to replicate results. Information on applying for ALSWH data is available from https://alswh.org.au/for-data-users/applying-for-data/. In addition, linked administrative data have been provided by the following third parties: • Australian Institute of Health and Welfare, Protocol EO2020/3/1115. • NSW Ministry of Health, Protocol 2019/ETH01837. • Victorian Department of Health, Protocol HREC/18/Austin/163. • Qld Health, Protocol 2019/ETH01837. • ACT Health, Protocol ETH.6.13.148. • The Department of Health Western Australia, Protocol RGS 4844. • ACT Health, Protocol ETH.6.13.148. • SA Health, Protocol ETH.6.13.148. • Northern Territory Department of Health, Protocol ETH 6.13.148. • The Department of Health Tasmania, Protocol H0017192. For these linked data to be accessed through ALSWH, every data user must be added to the applicable Data Use Agreements and Human Research Ethics Committee protocols.

## Abbreviations

AIC: Akaike information criterion
AIHW: Australian Institute of Health and Welfare
ALSWH: Australian Longitudinal Study on Women’s Health
ATC: Anatomic Therapeutic Chemical
AvePP: Average posterior probabilities
BIC: Schwarz Bayesian information criterion
BCH: Bolck Croon and Hagenaars
EM: Expectation–maximization
GP: General practitioner
IP: Inverse propensity
MBS: Medicare Benefits Schedule
PBS: Pharmaceutical Benefits Scheme
PIN: Person Identification Numbers
PMS: Premenstrual syndrome
