# Supplementary material for: Symptom patterns and health service use of women in early adulthood: a latent class analysis from the Australian Longitudinal Study on Women’s Health
Source: BMC Public Health. 2023 Jan 21;23:147. doi: 10.1186/s12889-023-15070-7 (PMC9863188; doi:10.1186/s12889-023-15070-7)
Supplement: Supplementary file 1 — Additional file 1: Appendix S1. The BCH approach and calculation of Inverse Propensity Weights. Figure S1. Covariate balance plot [standardised difference of BCH-weighted and BCH- and Inverse propensity weight-adjusted distribution] for Menstrual, Mood and Many Symptoms groups compared to Minimal Symptoms group. Table S1. BCH-weighted and BCH- and inverse propensity (IP) weighted percents of health case use for the whole study population and by symptom latent classes. [file 12889_2023_15070_MOESM1_ESM.pdf]

Supplementary Material

Symptom patterns and health service use of women in early adulthood : a latent class analysis from the Australian Longitudinal Study on Women's Health

Table of Contents

Appendix S1 The BCH approach and calculation of Inverse Propensity Weights 2

Figure S1 Covariate balance plot [standardised difference of BCH-weighted and BCH- and Inverse propensity weight-adjusted distribution] for Menstrual, Mood and Many Symptoms groups compared to Minimal Symptoms group. 3

Table S1 BCH-weighted and BCH- and inverse propensity (IP) weighted percents of health case use for the whole study population and by symptom latent classes. 4

References 5

Appendix S1 The BCH approach and calculation of Inverse Propensity Weights

To estimate the associations between covariates and latent class membership, we first used the Bolck Croon Hagenaars (BCH) method [1, 2] to take account of uncertainty of latent class assignment:

The BCH method of estimating the associations between covariates and latent class membership involves three steps:

1. The latent class model to define class membership is estimated using only the indicator variables; the covariates are not included in this model. The posterior probabilities of class membership are retained for each participant.
2. Participants are assigned to a latent class based on their highest posterior probability (modal assignment) and weights for each participant are calculated to account for classification error.
3. These weights are then used in the models for estimating multinomial regression coefficients for the associations between covariates (e.g., sociodemographic, health and behavioural variables) and latent class membership.

To account for imbalances of the covariates between the latent classes we calculated the Inverse Propensity (IP) weights for the analysis of the associations between latent class membership and health service use. We used the method described by Bray and colleagues [3]. The method has the following steps:

4. Using the β estimates (from Step 3 above), fitted probabilities of latent class membership for each participant are calculated conditional on the covariates, but not on the symptom (indicator) variables.
5. An estimated propensity score is then calculated for each participant. The propensity score is a weighted sum i.e., the sum of the probabilities calculated in step 4 weighted by the posterior probabilities of class membership (step 1).
6. The inverse propensity score for each participant is calculated as 1/propensity score.

We estimated the BCH- weighted and BCH- plus IP- weighted means and proportions for each potential confounder by latent class status. We then assessed the balance of the IP weights between the reference latent class and the other latent classes by comparing the standardised difference for each covariate.

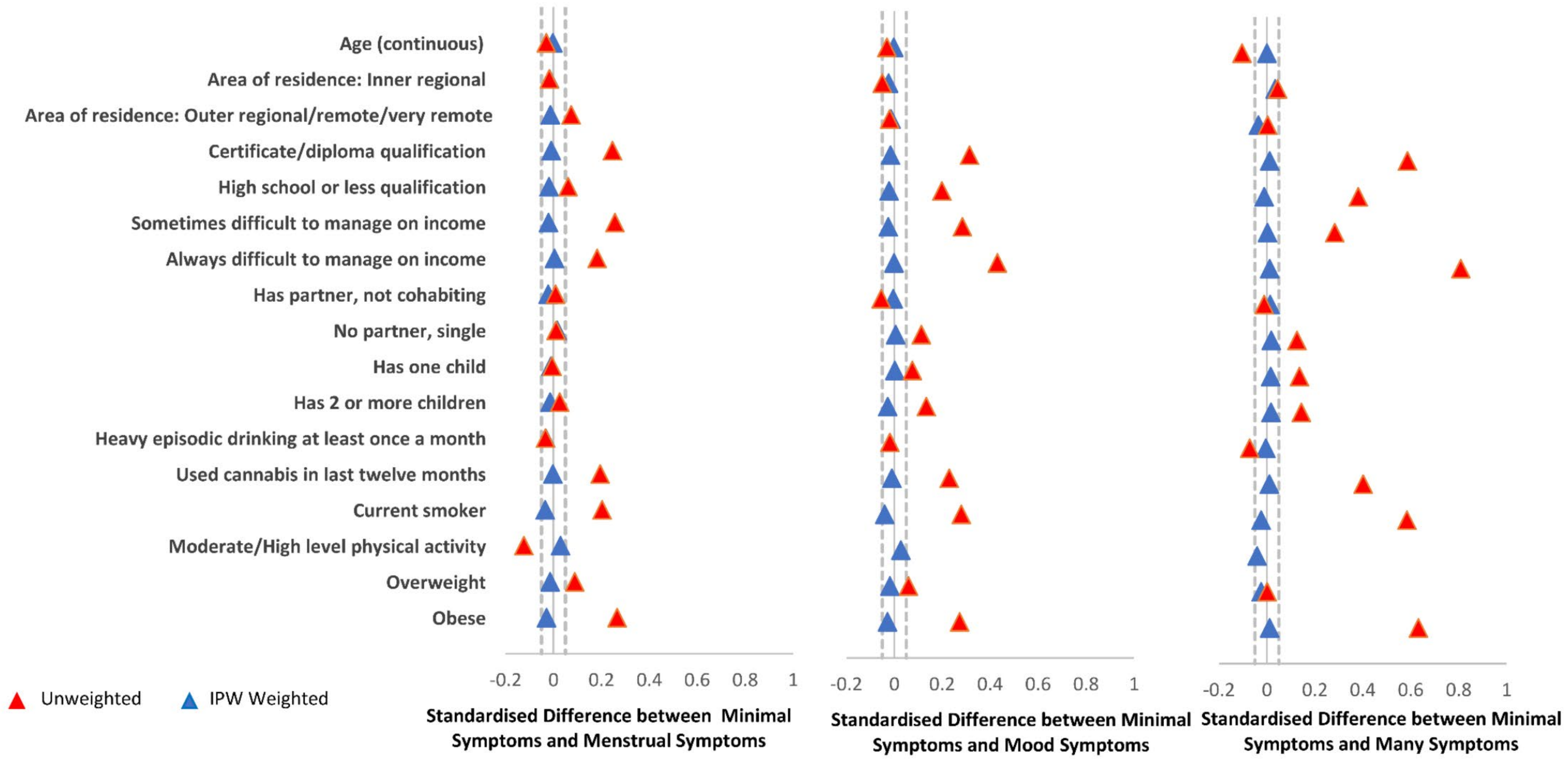

Figure S1 Covariate balance plot [standardised difference of BCH-weighted and BCH- and Inverse propensity weight-adjusted distribution] for Menstrual, Mood and Many Symptoms groups compared to Minimal Symptoms group.
Abbreviations: BCH = Bolck Croon Hagenaars

Table S1 BCH-weighted and BCH- and inverse propensity (IP) weighted percents of health case use for the whole study population and by symptom latent classes.

Health Service Use	Whole study population	Minimal Symptoms (36.6%)		Menstrual Symptoms (21.9%)		Mood Symptoms (26.2%)		Many Symptoms (15.3%)	
		BCH-adjusted	BCH-adjusted with IP weights	BCH-adjusted	BCH-adjusted with IP weights	BCH-adjusted	BCH-adjusted with IP weights	BCH-adjusted	BCH-adjusted with IP weights
GP Visits									
< 2 visits	16.1	21.0	20.2	17.0	16.8	11.6	11.7	10.6	12.3
2 to 3 visits	24.4	30.2	28.6	24.9	25.0	21.7	22.2	14.7	15.2
4 to 6 visits	28.9	29.1	29.6	29.5	29.2	29.8	30.0	25.9	26.9
7 to 9 visits	16.0	13.2	14.3	15.5	15.6	18.4	18.2	19.4	18.8
10 to 12 visits	7.8	4.6	5.1	7.9	8.2	9.0	8.8	13.3	12.4
> 12 visits	6.8	1.9	2.3	5.2	5.2	9.5	9.1	16.1	14.4
Specialist visits									
No visits	67.0	72.7	74.0	69.9	69.3	64.0	63.7	54.6	54.0
1 to 2 visits	20.2	19.5	18.3	19.8	19.8	20.4	21.2	22.0	23.0
≥ 3 visits	12.8	7.8	7.7	10.2	10.8	15.6	15.1	23.4	23.0
Prescribed medication use									
No medications	31.4	38.8	37.2	39.1	38.5	22.4	23.4	18.1	19.3
1 medication	28.2	32.4	32.1	26.0	25.5	27.8	27.8	21.9	22.9
2 medications	17.0	15.3	16.4	17.0	17.0	19.5	19.5	16.9	17.3
≥ 3 medications	23.4	13.4	14.3	17.9	18.9	30.4	29.4	43.1	40.5
Same day/overnight hospital admissions									
No admission	88.4	92.6	92.4	90.5	90.4	87.2	87.6	77.5	79.0
≥ 1 admission	11.6	7.4	7.6	9.5	9.6	12.8	12.4	22.5	21.0
Same day hospital admissions									
No admission	91.9	94.4	94.5	93.5	93.4	91.0	91.3	85.0	85.3
≥ 1 admission	8.1	5.6	5.5	6.5	6.6	9.0	8.7	15.0	14.7
Overnight hospital admissions									
No admission	94.7	97.3	97.1	96.0	96.1	94.1	94.3	87.3	89.0
≥ 1 admission	5.3	2.7	2.9	4.0	3.9	5.9	5.7	12.7	11.0

ABBREVIATIONS: BCH = Bolck Croon Hagenaars; IP = inverse propensity

References

1. Bolck A, Croon M, Hagenaars J. Estimating latent structure models with categorical variables: One-step versus three-step estimators. Polit Anal 2004, 12(1):3-27. https://doi.org/10.1093/pan/mph001

2. Dziak JJ, Bray BC, Wagner AT. LCA_Covariates_3Step SAS macro users' guide (Version 1.0). University Park, PA: The Methodology Center, Penn State. Retrieved from http://methodology.psu.edu 2020.

3. Bray BC, Dziak JJ, Patrick ME, Lanza ST. Inverse Propensity Score Weighting with a Latent Class Exposure: Estimating the Causal Effect of Reported Reasons for Alcohol Use on Problem Alcohol Use 16 Years Later. Prev Sci 2019, 20(3):394-406. https://doi.org/10.1007/s11121-018-0883-8
